# An online database for brain disease research

**DOI:** 10.1186/1471-2164-7-70

**Published:** 2006-04-04

**Authors:** Brandon W Higgs, Michael Elashoff, Sam Richman, Beata Barci

**Affiliations:** 1 Elashoff Consulting LLC, Germantown, MD 20876, USA; 2 Stanley Medical Research Institute, Bethesda, MD, 20814-2142, USA

## Abstract

**Background:**

The Stanley Medical Research Institute online genomics database (SMRIDB) is a comprehensive web-based system for understanding the genetic effects of human brain disease (i.e. bipolar, schizophrenia, and depression). This database contains fully annotated clinical metadata and gene expression patterns generated within 12 controlled studies across 6 different microarray platforms.

**Description:**

A thorough collection of gene expression summaries are provided, inclusive of patient demographics, disease subclasses, regulated biological pathways, and functional classifications.

**Conclusion:**

The combination of database content, structure, and query speed offers researchers an efficient tool for data mining of brain disease complete with information such as: cross-platform comparisons, biomarkers elucidation for target discovery, and lifestyle/demographic associations to brain diseases.

## Background

Brain disease studies based on experiments using genome-wide measurements with microarrays are traditionally challenging as compared to other disease areas. The biological results are often hindered by statistical issues of small sample sizes, small effect sizes, and patient-to-patient variability [1-3]. Also, clinical information for patients is typically sparse, such that unknown clinical covariates can either confound or confuse many of the gene expression patterns and trends, as opposed to the primary disease. Corrections using such clinical information can greatly improve inference in determining markers for disease, as well as elucidating patterns within the disease.

Technical problems in microarray data can also affect the analyses. Meaningful results are often limited by array platform-to-platform comparisons and overall organization/presentation of large data sets/results. Studies conducted on disparate platforms are inherently more difficult to analyze than those conducted on the same platform [4]. Cross-platform comparisons present analysis challenges due to differences in scaling and sensitivity (to name a few) which introduce inconsistencies in reproducibility [5-8]. Large data sets and comprehensive results summaries present another challenge that requires good organization of both analytical and bioinformatics information (e.g. expression profiles, gene summary information, pathway diagrams, fold change value comparisons, etc.) into a user-friendly format to facilitate efficient data mining. A relational web-based tool that logically combines all of these factors can enhance researchers' ability to determine the underlying genomic patterns in brain disease.

The SMRIDB is an online data warehouse and analytical system designed to aid researchers in understanding the biological associations both between and within the brain disorders of schizophrenia, bipolar, and major depression. This open source database combines genomic patterns of brain disease with patient clinical metadata into a user-friendly query interface to enable efficient data mining for purposes of biomarker discovery and elucidating biological mechanisms of brain disease. The metadata includes a full summary of clinical history for each patient with hyperlinks to disease-level information, such that demographic- and lifestyle-associated effects can be determined as they relate to brain disorders. The genomic data has been compiled from 12 separate labs (identified as studies), each data set generated from brain tissue isolated from two controlled populations of 165 patients, diagnosed with one of the three brain disorders (plus unaffected control brain tissue). This genomic data has been generated across 6 separate human array platforms (Affymetrix: hgu133a, hgu133plus, hgu95av2, Agilent, Codelink, and cDNA custom array) providing patterns/trends and analytical inferences that are not limited by platform dependencies.

## Construction and content

### Bioinformatics mappings

NCBI's Database for Annotation, Visualization and Integrated Discovery (DAVID 2.0) was used as the standard source for gene annotation information [9]. The primary fields extracted from DAVID include: LocusLink, gene symbol, and gene summary. Additional annotations include gene product mappings to the Kyoto Encyclopedia of Genes and Genomes (KEGG), and Gene Ontology Consortium (GO) for pathway and GO terms/classes, respectively. For Affymetrix arrays, queries were based on the Affymetrix probe ID (AFFYID). For other arrays, the Genbank accessions (GENBANK) were used.

### Individual study-level analysis

**Figure 1:**
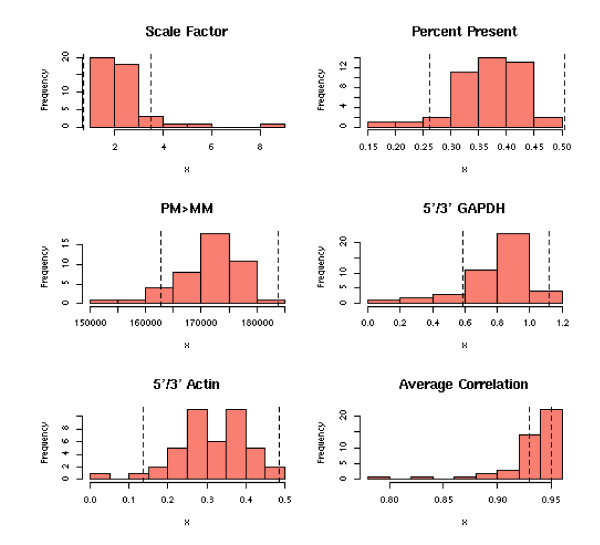
**QC histograms**. Examples of distribution thresholds used to assess outliers for an individual study.

**Figure 2:**
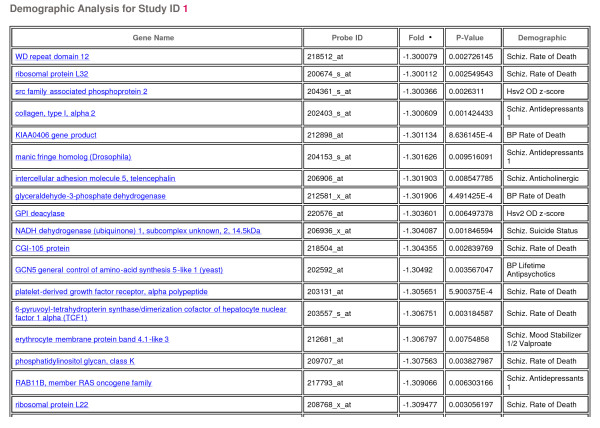
**Demographic gene table**. Table of genes determined to be significant (p < 0.01 and fold change > 1.3) with the demographic variables for an individual study.

For each of the individual studies, a series of analyses were performed. Each array (representing a single patient) was subjected to a quality control (QC) analysis for chip-level parameters (e.g. scaling factor, gene calls, control gene ratios, average correlation) with respect to the reference distribution for those parameters across the arrays. This QC analysis is represented with both graphical representations (e.g. heatmaps, scatter plots, and histograms (Figure 1)) and table summaries, allowing users to readily identify those arrays determined to be outliers in the study. A total of 41 clinical demographic variables (Tables 1, 2, 3, 4) were assessed for their effects on a gene-by-gene basis. Continuous variables and ordered categorical variables were cut at values as close as possible to the median (e.g. PMI>30 vs. PMI<30; Drug Use = 'Heavy' vs. Drug Use = 'None, Light, Moderate'). The genes determined to be most significant (p-value<0.01 and fold change >1.3) for each demographic variable is reported in a table, accompanied by a summary of the percentage of significant genes for each variable (Figure 2). Each gene found to be significant for a demographic variable links to a gene-centric page (discussed in **Gene details page **section). Such results allow researchers to determine markers that are related to lifestyle or clinical demographical information and identify confounding variables within a disease class.

**Table 1:** Patient demographic variables for all diseases

**All Patient Variables**	**Values**
Age >45	Yes/No
PMI > 30	Yes/No
Brain pH > 6.5	Yes/No
Left Brain	Yes/No
Sex	Male/Female
Smoking at Time of Death	Yes/No
Herpes simplex virus 1 OD	High/Low
Herpes simplex virus 2 OD	High/Low
Toxolgg OD	High/Low
EBV OD	High/Low
HHV6 OD	High/Low
CMV OD	High/Low
Hervk 18 SNP	Positive/Negative
Hervk 18 Expression	Positive/Negative

**Table 2:** Patient demographic variables for Bipolar patients

**Bipolar Patient Variables**	**Values**
Bipolar Severity	Severe/Other
Bipolar Heavy Alcohol Use	Yes/No
Bipolar Heavy Drug Use	Yes/No
Bipolar Psychotic Feature	Yes/No
Bipolar Sudden Death	Yes/No
Bipolar Suicide Status	Yes/No
Bipolar Lifetime Antipsychotics >0	Yes/No
Bipolar Antipsychotics	Yes/No
Bipolar Antidepressants	Yes/No
Bipolar Mood Stabilizer	Yes/No
Bipolar Lithium	Yes/No
Bipolar Valproate	Yes/No

**Table 3:** Patient demographic variables for Schizophrenic patients

**Schizophrenic Patient Variables**	**Values**
Schizophrenia Severity	Severe/Other
Schizophrenia Heavy Alcohol Use	Yes/No
Schizophrenia Heavy Drug Use	Yes/No
Schizophrenia	Paranoid/Undiff
Schizophrenia Sudden Death	Yes/No
Schizophrenia Suicide	Yes/No
Schizophrenia Lifetime Antipsychotics >45,000	Yes/No
Schizophrenia Antichollnergic	Yes/No
Schizophrenia Antidepressants	Yes/No
Schizophrenia Stabilizer	Yes/No
Schizophrenia Lithium	Yes/No
Schizophrenia Valproate	Yes/No

**Table 4:** Patient demographic variables for Depressed patients

**Depressed Patient Variables**	**Values**
Depression Heavy Alcohol Use	Yes/No
Depression Heavy Drug Use	Yes/No
Depression Suicide	Yes/No

**Figure 3:**
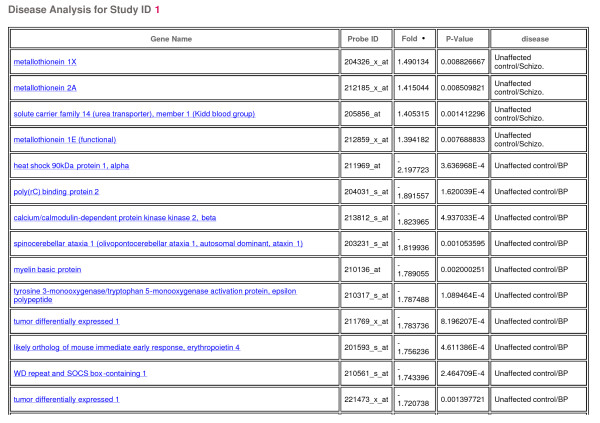
**Disease gene table**. Table of genes determined to be significant (p < 0.01 and fold change > 1.3) with the disease for an individual study.

**Figure 4:**
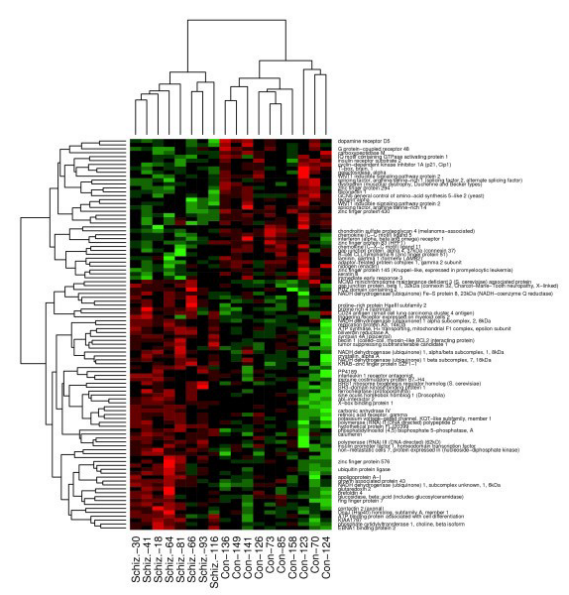
**Study-level visuals (heatmap)**. Two-dimensional hierarchical clustering heatmap containing the most significant genes in schizophrenic disease for an individual study.

**Figure 5:**
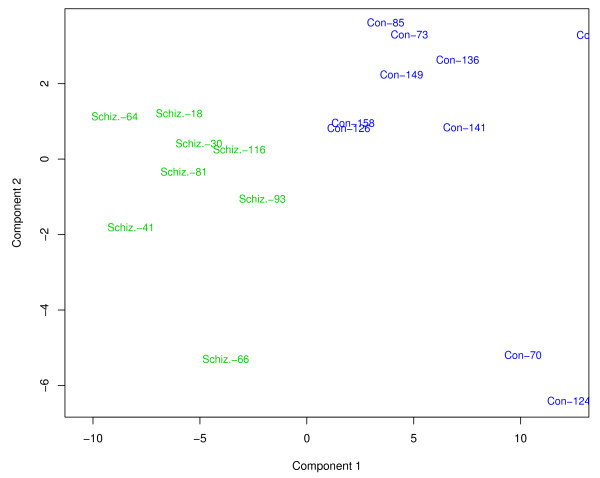
**Study-level visuals (PCA scatter plot)**. Principal components plots generated with the most significant genes in schizophrenic disease for an individual study.

**Figure 6:**
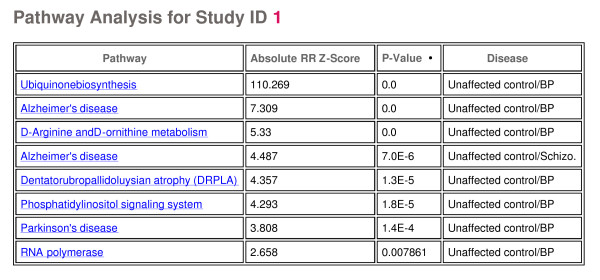
**Pathway table**. Table of most regulated pathways for an individual study.

The three disease classes were analyzed to provide a list of discriminating genes (adjusted for the demographic terms that met the criteria of significance for that gene) or markers indicative of disease (Figure 3) between the control patients and each disease class (schizophrenia, bipolar, depression). In addition to table summaries (genes in table also link to their respective gene detail page), both 2D clustering heatmaps (Figure 4) and principal components scatter plots (Figure 5) are provided for a visual representation of the data. Utilizing these disease markers, the most regulated pathways and GO terms were identified for each disease comparison based on a Fisher's exact test. Each pathway and GO term (from each of the three GO functional classifications separately) is ranked by p-value for each disease comparison to indicate the most regulated pathway/GO terms (Figure 6). Additionally, each pathway and GO term in the table links to a pathway/GO detail page.

### Pathway/GO details page

**Figure 7:**
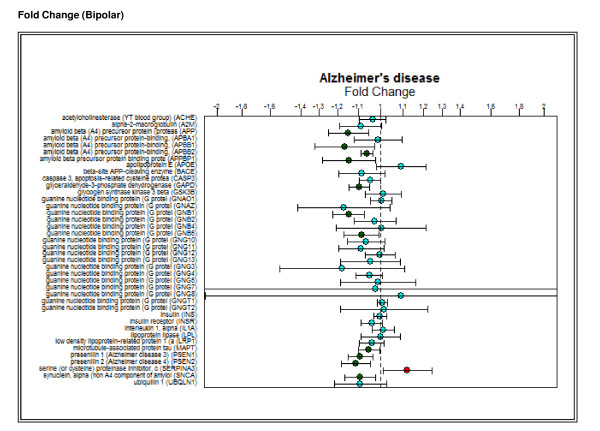
**Fold change boxplots**. Fold change (with confidence intervals) values for bipolar patients for every gene that maps to the Alzheimer's pathway.

Within this pathway/GO detail page is a comprehensive summary of the gene expression profiles for each gene that is mapped to the associated pathway or GO term within each separate disease class. A confidence interval boxplot is provided within each disease comparison inclusive of every gene mapped to that pathway or GO term queried in the study (Figure 7), along with a link to the pathway network representation provided by KEGG. Such results allow researchers to understand the most regulated biological mechanisms and cellular sites for each disease class.

### Gene details page

**Figure 8:**
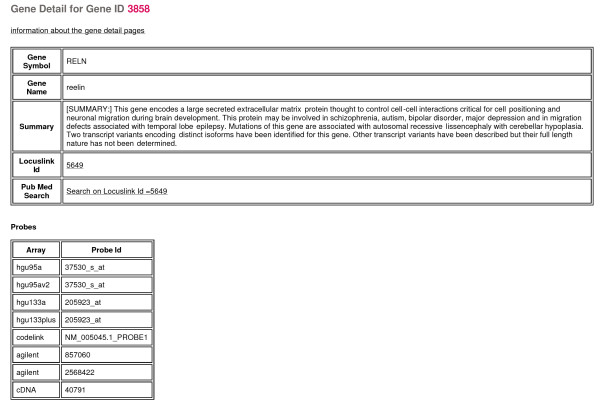
**Gene summary page (truncated)**. Portion of gene summary page for the gene reelin (RELN).

**Figure 9:**
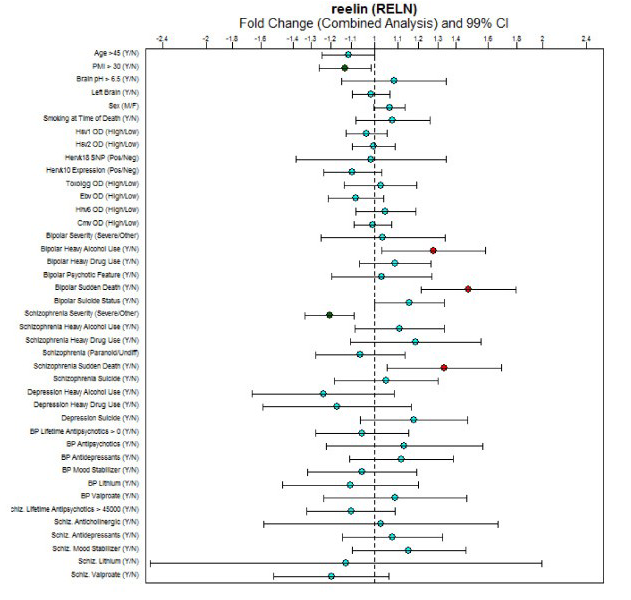
**Fold change boxplots**. Fold change (with 99% confidence intervals) for the gene reelin across all 41 demographic variables.

For every probe across the 6 array platforms, primary annotations were determined such that each probe is mapped to either a gene name or EST identifier (refer to **Bioinformatics mappings **section for mapping criteria). So each gene summary page contains probe-level information for all of the 6 array platforms and 12 studies within the database. In addition to general bioinformatics annotations (e.g. biological summary, LocusLink ID, PubMed search link, and gene symbol) and pathway/GO mappings (associations with gene that link to pathway/GO-centric pages), this page contains gene expression summaries for every probe that maps to this gene across all studies (Figure 8). A cross study 'consensus' fold change was calculated for each gene and disease/demographic comparison, based on a weighted combination of the individual fold changes and standard errors for the probes that map to each gene across the platforms/studies. Weights were determined in a probeset-specific manner to account for the differing levels of precision associated with each probeset that maps to a given gene across the platforms. Confidence interval boxplots inclusive of each probe for the gene on this page are provided for the following: normalized expression across all patients, fold changes within each disease class, percent present calls for the former two comparisons, and all 41 demographic variables for the gene (Figure 9). Additionally, there is a general search engine that supports queries of gene name, symbol, pathway, GO term, and LocusLink ID designed for direct access to any gene detail page or pathway/GO detail page.

### Cross-platform analysis

To date, making comparisons across disparate gene expression platforms has been very difficult [5-8]. Chip manufacturing differences such as probe selection, processing protocols, and spot normalization algorithms contribute to variability that can distort mRNA transcript abundance measurements and introduce inconsistencies to hinder cross-platform comparisons. Some success has been demonstrated in reducing the problem to the most consistent sequence-verified gene annotations between two platforms (e.g. UniGene cluster membership) and examining correlations, ratio values, or gene calls, although sensitivity and global statistical inference of such approaches still remains a challenge [7,10-12].

**Figure 10:**
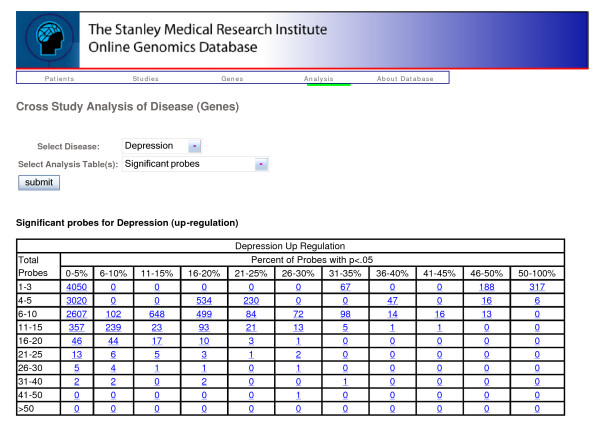
**Summary statistic table**. Gene-level summary table of significant probes across all studies for depression.

**Figure 11:**
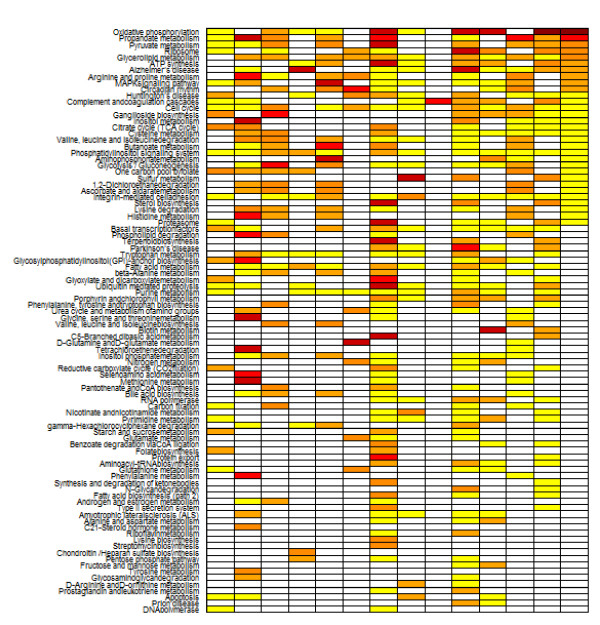
**Pathway clickable heatmap**. Study-centric clickable heatmap of top regulated pathways in schizophrenia. Each column can be sorted by a particular study or the three last summary columns. Study 12 was omitted from this visual.

**Figure 12:**
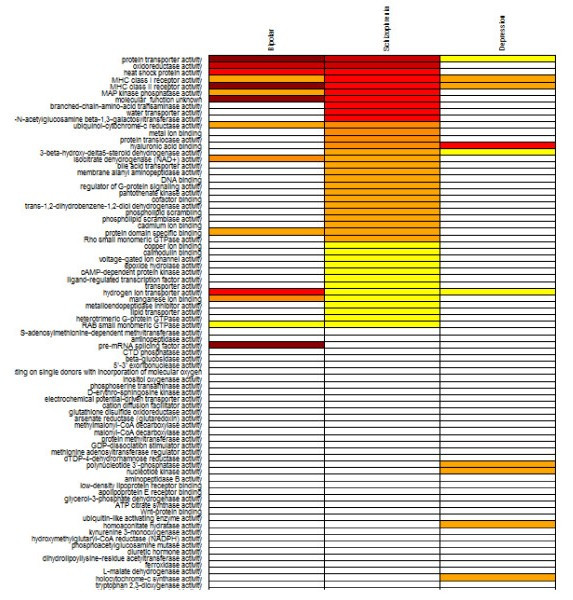
**GO term clickable heatmap**. Gene-centric clickable heatmap of top regulated GO terms (molecular function) in schizophrenia. Each column can be sorted by a disease.

**Figure 13:**
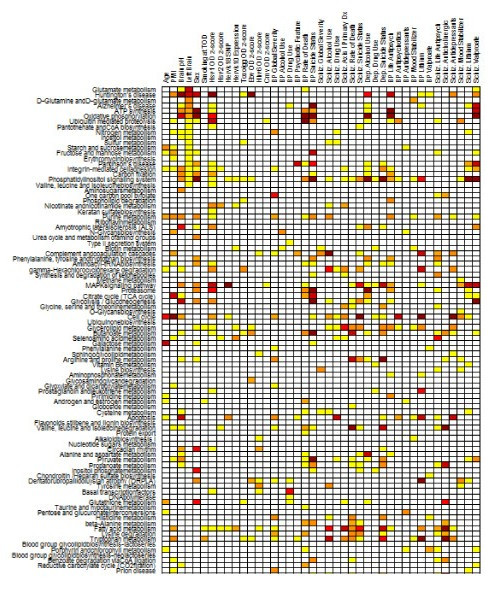
**Pathway/demographic clickable heatmap**. Demographic variable clickable heatmap of top regulated pathways. Each column can be sorted by a demographic variable.

The cross-platform comparisons within the SMRIDB are based on scaled representations of individual study-level analysis across studies to extract biological patterns and relationships. These cross-platform results are provided for both the gene level (Figure 10) and pathway/GO level in a study-centric (Figure 11) and gene-centric (Figure 12) visualization. For the gene-level cross-platform analysis, the fold changes and confidence intervals are calculated as described in the **Gene details page **section. For the pathway/GO-level analysis, the p-values calculated by the Fishers's exact test from each study individually for disease-related genes were scaled across studies and provided in an interactive sortable heatmap, where each cell has a clickable link to a pathway/GO details page. Additionally, this same analysis and visual representation is provided for the demographic variables (Figure 13). Such a data representation allows researchers to quickly determine the most regulated pathways or functional classifications across all platforms or for a specific demographic variable.

## Utility and discussion

The user interface was constructed to enable intuitive navigating and efficient data mining. The main site contains the primary index for the database's 4 general segmented areas: Patients, Studies, Genes, and Analysis, each of which is a gateway to unique focus areas, with mutual associations between each, such as clinical information vs. genomics results and individual study content vs. cross-platform combined analyses. The Genes tab contains an open text search engine (with partial matches) to enable queries by gene, LocusLink, or pathway for any single or combined study results.

The intended users of the database include any genomics researchers facing the persistent challenges of sensitivity for biomarker discovery and cross-platform microarray comparisons. However, the content within the SMRIDB is primarily designed for biologists, clinical researchers, bioinformaticians, and scientist in the field of brain disease.

The size and scope of the SMRIDB makes it a unique contribution to genomics-based brain disease research. With combined gene expression profile summaries across 12 studies and 6 platforms, there is greater confidence in scientific findings such as biomarkers for disease, biological functional roles, and regulated pathways, as compared to results obtained from any one individual study.

## Conclusion

The SMRIDB is a comprehensive data mining tool to enable researchers to elucidate the biological mechanisms of bipolar disorder, schizophrenia, and depression. A diverse patient population combine with data generated across six microarray platforms and 12 studies to provide robust results to enhance the understanding of brain disease.

## Availability and requirements

The SMRIDB can be accessed at . All users must register (name and email address) to obtain a username and password.

## Authors' contributions

BWH and ME conducted the data analysis and were involved in drafting the manuscript. SR developed the web services and database backend. BB collected and catalogued the clinical information and samples. All authors read and approved the final manuscript.
